# AtHDA6 functions as an H3K18ac eraser to maintain pericentromeric CHG methylation in *Arabidopsis thaliana*

**DOI:** 10.1093/nar/gkab706

**Published:** 2021-08-17

**Authors:** Qianwen Wang, Xiucong Bao, Shengjie Chen, Huan Zhong, Yaqin Liu, Li Zhang, Yiji Xia, Friedrich Kragler, Ming Luo, Xiang David Li, Hon-Ming Lam, Shoudong Zhang

**Affiliations:** School of Life Sciences, The Chinese University of Hong Kong, Shatin, Hong Kong Special Administrative Region; Center for Soybean Research of the State Key Laboratory of Agrobiotechnology, The Chinese University of Hong Kong, Shatin, Hong Kong Special Administrative Region; Department of Chemistry, The University of Hong Kong, Pokfulam Road, Hong Kong Special Administrative Region; School of Life Sciences, The Chinese University of Hong Kong, Shatin, Hong Kong Special Administrative Region; Center for Soybean Research of the State Key Laboratory of Agrobiotechnology, The Chinese University of Hong Kong, Shatin, Hong Kong Special Administrative Region; Department of Biology, Hong Kong Baptist University, Kowloon, Hong Kong Special Administrative Region; School of Life Sciences, The Chinese University of Hong Kong, Shatin, Hong Kong Special Administrative Region; Center for Soybean Research of the State Key Laboratory of Agrobiotechnology, The Chinese University of Hong Kong, Shatin, Hong Kong Special Administrative Region; School of Life Sciences, The Chinese University of Hong Kong, Shatin, Hong Kong Special Administrative Region; Center for Soybean Research of the State Key Laboratory of Agrobiotechnology, The Chinese University of Hong Kong, Shatin, Hong Kong Special Administrative Region; Center for Soybean Research of the State Key Laboratory of Agrobiotechnology, The Chinese University of Hong Kong, Shatin, Hong Kong Special Administrative Region; Department of Biology, Hong Kong Baptist University, Kowloon, Hong Kong Special Administrative Region; State Key Laboratory of Environmental and Biological Analysis, Hong Kong Baptist University, Kowloon, Hong Kong Special Administrative Region; Max-Planck-Institute of Molecular Plant Physiology, Wissenschaftspark Golm, Am Mühlenberg 1, 14476 Golm, Germany; Agriculture and Biotechnology Research Center, Guangdong Provincial Key Laboratory of Applied Botany, Center of Economic Botany, Core Botanical Gardens, South China Botanical Garden, Chinese Academy of Sciences, Guangzhou 510650, China; Department of Chemistry, The University of Hong Kong, Pokfulam Road, Hong Kong Special Administrative Region; School of Life Sciences, The Chinese University of Hong Kong, Shatin, Hong Kong Special Administrative Region; Center for Soybean Research of the State Key Laboratory of Agrobiotechnology, The Chinese University of Hong Kong, Shatin, Hong Kong Special Administrative Region; School of Life Sciences, The Chinese University of Hong Kong, Shatin, Hong Kong Special Administrative Region; Center for Soybean Research of the State Key Laboratory of Agrobiotechnology, The Chinese University of Hong Kong, Shatin, Hong Kong Special Administrative Region

## Abstract

Pericentromeric DNA, consisting of high-copy-number tandem repeats and transposable elements, is normally silenced through DNA methylation and histone modifications to maintain chromosomal integrity and stability. Although histone deacetylase 6 (HDA6) has been known to participate in pericentromeric silencing, the mechanism is still yet unclear. Here, using whole genome bisulfite sequencing (WGBS) and chromatin immunoprecipitation-sequencing (ChIP-Seq), we mapped the genome-wide patterns of differential DNA methylation and histone H3 lysine 18 acetylation (H3K18ac) in wild-type and *hda6* mutant strains. Results show pericentromeric CHG hypomethylation in *hda6* mutants was mediated by DNA demethylases, not by DNA methyltransferases as previously thought. DNA demethylases can recognize H3K18ac mark and then be recruited to the chromatin. Using biochemical assays, we found that HDA6 could function as an ‘eraser’ enzyme for H3K18ac mark to prevent DNA demethylation. Oxford Nanopore Technology Direct RNA Sequencing (ONT DRS) also revealed that *hda6* mutants with H3K18ac accumulation and CHG hypomethylation were shown to have transcriptionally active pericentromeric DNA.

## INTRODUCTION

**Figure 1.**
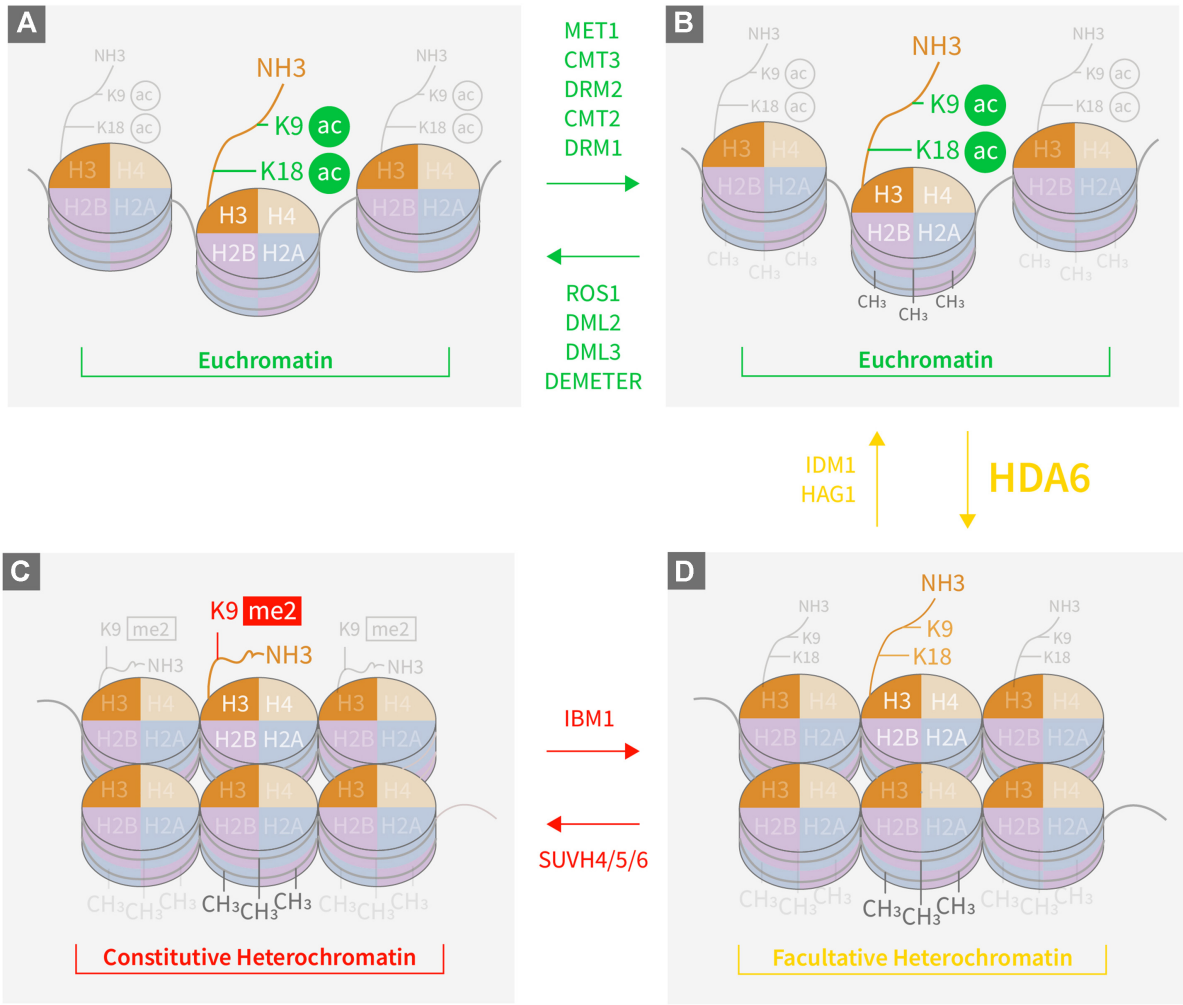
Schematic representation of chromatin dynamics mediated by DNA methylation and histone modifications. (**A**) Euchromatin, with H3K9ac and H3K18ac histone marks and minimal levels of methylated DNA after catalysis by DNA demethylases (in green above right-pointing arrow). (**B**) Euchromatin, with relatively high levels of methylated DNA (shown as light and dark grey –CH3 groups) catalyzed by DNA methyltransferases (in green below left-pointing arrow), or transitioned from facultative heterochromatin after catalysis by IDM1 and HAG1. (**C**) Facultative heterochromatin, transitioned from highly methylated euchromatin after catalysis by HDA6, or transitioned from constitutive heterochromatin after IBM1 catalysis. (**D**) Constitutive heterochromatin, transitioned from facultative heterochromatin after catalysis by SUVH4/5/6.

Epigenetic modifications, including DNA methylation and histone modifications, are reversible processes that play crucial roles in maintaining genomic integrity and stability while modulating gene expression (Figure 1) (1–3). This allows eukaryotic cells to adjust their chromatin status in response to endogenous and exogenous stimuli (4,5). Gene expression is activated in open chromatin (euchromatin), which is determined by active epigenetic marks, but blocked by repressive epigenetic marks in heterochromatin (6). Both cytosine-based DNA methylation and histone H3 lysine 9 dimethylation (H3K9me2) are regarded as key repressive marks, particularly at constitutive heterochromatin regions in *Arabidopsis thaliana* (7). DNA can be methylated by DNA methyltransferases such as MET1 (METHYLTRANSFERASE 1) for CG methylation, CMT3 (CHROMOMETHYLASE 3) for CHG methylation, and DRM2 (DOMAINS REARRANGED METHYLTRANSFERASE 2) for CHH methylation (1). DNA demethylation, on the other hand, requires DNA demethylases, such as ROS1 (REPRESSOR OF SILENCING 1), DME (DEMETER), DML2 (DEMETER-LIKE 2) and DML3 (DEMETER-LIKE 3), to remove methyl groups from cytosines (Figure 1) (4). Demethylation by DME occurs in heterochromatin regions in central cells (8,9) and in vegetative tissues (10) in Arabidopsis. The maintenance of DNA methylation status, particularly in the CHG context in constitutive heterochromatin, requires H3K9me2 repressive mark, whose addition and removal are catalyzed by methyltransferases SUVH4/5/6 (SUPPRESSOR OF VARIEGATION 4/5/6) (11), and IBM1 (INCREASE IN BONSAI METHYLATION 1) (12), respectively. Our previous observation that mutations in histone deacetylase 6 (HDA6) could reverse the DNA demethylation-deficient phenotype of *ros1-1* mutants (13,14) led us to hypothesize that DNA methylation could be regulated by histone deacetylation via HDA6.

It is known that the H3K18ac mark is a prerequisite for DNA demethylation (15,16). For DNA demethylases to access methylated DNA, the histone mark H3K18 must first be acetylated by IDM1 (16) via a complex consisting of MBD7 (methyl-CpG-binding domain-containing protein 7), IDM2 and IDM3 (17,18). Deacetylation of H3K18ac is also required to maintain the genome-wide DNA methylation status, which inhibits euchromatin spreading and silence potentially harmful genomic regions such as transposons and repeat sequences. Although the writer enzyme that acetylates H3K18 has been identified (15,19), the H3K18ac eraser remains unknown. In this study, we show that HDA6 acts as the H3K18ac eraser, thereby antagonizing the acetyltransferase activity of IDM1 and blocking the access of DNA demethylases to their target DNA. Thus, HDA6 functions to maintain the DNA methylation marks (Figure 1).

## MATERIALS AND METHODS

### Plant materials

Arabidopsis ecotype C24 (wild type background), *ros1-1*, *ros1-1&hda6-9* and *ros1-1&hda6-10* were used in the study (13). Seeds were surface-sterilized with 50% commercial bleach and 0.1% Triton-X100 for 10 min, and then washed five times with sterilized ddH_2_O. The seeds were then sowed on $\frac{1}{2}$ MS (Murashige and Skoog) medium supplemented with 1% sucrose in Petri dishes. Each Petri dish was divided into four quadrants, each for seeds of a different genotype. The sowed seeds were then stratified at 4°C for 4 days. After stratification, the seeds were grown in a 16 h/8 h light/dark cycle at 22°C for 12 days. Then the seedlings were harvested for genomic DNA extraction and bisulphite sequencing or for ChIP-seq experiments. The transgenic Arabidopsis plants harboring full-length HDA6-3xFLAG were acquired via BP cloning by introducing the full-length *HDA6* coding region into the pENTRY/D-TOPO vector. LR reaction was then employed to clone targeted DNA fragment into the binary vector pEarley101-YFP-3Flag. After transforming the recombinant vector into the Agrobacterium GV3101, floral dipping was used to produce transgenic Arabidopsis expressing HDA6-YFP-3FLAG. The T3 transgenic seeds were grown in liquid $\frac{1}{2}$ MS medium supplemented with 1% sucrose, with shaking at 120 rpm in a 16h/8h light/dark cycle at 22°C for 2 weeks to get enough plant materials for Co-IP to purify the 3XFLAG-tagged HDA6 proteins from the transgenic Arabidopsis seedlings.

### Whole genome bisulfite sequencing (WGBS) and analysis

Genomic DNA was extracted from 12-day-old seedlings of Arabidopsis wild type and mutants using GeneJET plant genomic DNA purification mini kit (Thermo Fischer Scientific, Cat# K0792). The purified genomic DNA were subjected to whole-genome bisulfite sequencing (WGBS) on the DNBseq™ platform (BGI company). Each sample was spiked with Lamda DNA to calculate the conversion ratio, and each sample was sequenced with two biological replicates.

The WGBS data for Col-0 genetic background of the mutants used were downloaded from the GEO database (accession no. GSE39901 for the BS-seq of *hda6-6*, *hda6-7*, Col-0 for *hda6*, *SUVH4/5/6* and Col-0 (20); accession no. GSE83802 for WGBS data of *ros1-4* and Col-0 (21); accession no. GSE164916 for the WGBS data of *drdd^DD7 pro^* and Col-0 (10)).

For data analysis, the adapter and low-quality sequences were trimmed using TrimGalore (https://github.com/FelixKrueger/TrimGalore) with these parameters: three_prime_clip_R1 3; three_prime_clip_R2 3; clip_R1 2; clip_R2 2; max_n 10; trim-n. The bismark (22) was then used to align the clean sequences of the samples in either the C24 or the Col-0 background to the published C24 (23) or TAIR10 (Col-0) (24) genome, respectively. The uniquely mapped sequences were then extracted using bismark-deduplicated (22). The cytosines with 5x coverage in all libraries in the same background were visualized in Jbrowse (25) by jbplugin-methylation (26) and the differentially methylated loci (DMLs) were identified via DMLtest in the DSS R package (*P*-values ≤ 0.05) (27). The DML results were then visualized by Integrated Genomics Viewer (IGV) (28). To detect differentially methylated regions (DMRs), CallDMR (27) was used, with the minimum length set at 100 bp and minimum number of sites set at 4. For the samples in the C24 background, the final DMRs were filtered by the methylation difference of 0.4, 0.25 and 0.15 for the CG, CHG and CHH contexts, respectively, while the DMR cut-off for the CG, CHG and CHH contexts in the Col-0 background were set at 0.3, 0.2 and 0.1, respectively. The genome-wide distribution of final DMRs was visualized by a circos plot (29). The three or four k-means clustering was applied to the different contexts of DMRs. The genomic location of a DMR was defined as follows: transposon (TE): from the transposon start site to transposon stop site; gene: from the transcription start site (TSS) to transcription termination site (TES); promoter: from TSS to 2 kb upstream; and intergenic regions: any genomic regions other than the above-defined categories. Each DMR was assigned to one category only and in the order listed above.

### Chromatin immunoprecipitation sequencing (ChIP-seq) and analysis

ChIP-seq was performed as previously reported with minor modifications (30). Twelve-day-old Arabidopsis seedlings were collected and crosslinked in 1% formaldehyde 10 min at room temperature. Nuclei were then purified from the cross-linked tissue and sonicated to generate genomic DNA fragments of about 200–500 bp. ChIP was performed using anti-H3K18ac antibodies (Millipore Sigma, Cat# 07-354). After de-crosslinking and DNA purification following the published protocol (30), a DNA library was prepared by using the VAHTS Universal DNA Library Prep Kit (Vazyme, ND607-01). Finally, the library was sequenced on the Illumina HiSeq X-Ten platform (Annoroad Gene Technology company).

Raw sequences were trimmed using TrimGalore to discard adapter and low-quality sequences (https://github.com/FelixKrueger/TrimGalore) with these parameters: three_prime_clip_R1 3; three_prime_clip_R2 3; clip_R1 12; clip_R2 12; max_n 10; trim-n. The clean sequences were then aligned to the Arabidopsis C24 genome (23) by Bowtie2 (31) with default parameters. Only uniquely mapped sequences were extracted for downstream analyses. To identify the peaks that were significantly enriched in the pairwise comparisons, diffreps (32) was used, with these parameters: *P*-value: 0.01; windows: 200; fold change cut-off: 2. deepTool (33) and BEDtools (34) were used to convert the peaks to the bed format and the results were visualized using circos plots (29) and JBrwose (25). The H3K18ac levels at the DMR loci were then extract by deepTools (33) and BEDTools (34).

### HDA6 protein expression and purification

To construct recombinant His-tagged HDA6 cDNA and its mutant versions, cDNAs of the full-length *HDA6* and its mutated version *HDA6-9* were amplified via RT-PCR with total RNAs of the wild type and the mutant *ros1-1&hda6-9*, respectively. The primers containing EcoRI and HindIII restriction sequences can be found in Supplementary Table S2. The amplified cDNAs were digested with EcoRI and HindIII, and then ligated into the pET32A vector digested with the same enzymes. The proteins were expressed in *Escherichia coli* Rosetta cells. To induce the expression of the target proteins, isopropyl β-d-1-thiogalactopyranoside (IPTG) was added to a final concentration of 0.2 mM when OD_600_ of the bacterial culture reached 0.6, and then the culture was grown at 16°C for another 16–18 h. Cells were harvested and resuspended in a lysis buffer (50 mM Tris–HCl, pH 8, 500 mM NaCl, 10% glycerol, 1 mM phenylmethanesulfonylfluoride (PMSF), and EDTA-free protease inhibitors [Roche, Cat# 4693132001]). The cell suspension was sonicated with 10 short bursts of 30 sec followed by intervals of 30 s for cooling (Note: keep the suspension on ice). Cell debris was removed by ultracentrifugation at 4°C for 30 min at 20 000 × g, and then the supernatant was loaded onto a nickel column (ThermoFisher Scientific, Cat# 88221) pre-equilibrated with the lysis buffer. The column was washed with 5 column volumes of a wash buffer (lysis buffer supplemented with 30 mM imidazole) and then the target proteins were eluted with an elution buffer (lysis buffer supplemented with 250 mM imidazole). The proteins were further purified by a HiLoad 26/60 Superdex 200 gel filtration column (GE Healthcare Life Sciences, United Kingdom, Cat# 28-9893-36) and concentrated with a Mono Q 5/50 GL column (GE Healthcare Life Sciences, United Kingdom, Cat# 17-5166-01). After concentration, the target proteins were stored at −80°C.

### Peptide synthesis and purification

All peptides were synthesized on the Rink-Amide MBHA resin following a standard Fmoc-based solid phase peptide synthesis protocol (35). Removal of protecting groups and cleavage of peptides from the resin were done by incubating the resin with a cleavage cocktail containing 95% trifluoroacetic acid (TFA), 2.5% triisopropylsilane, 1.5% water, and 1% thioanisole for 2 h at room temperature. The resulting peptides were purified by preparative HPLC with an XBridge Prep OBD C18 column (30 mm × 250 mm, 10 μm; Waters, Cat# 186003897). The purity and identity of the peptides were verified by LC–MS. The peptide sequences can be found in Supplementary Table S3.

### *In-vitro* enzymatic activity assay for HDA6

The enzymatic activities of HDA6 were measured by detecting the removal of the acetyl group from peptides. HDA6 proteins (2.5 μM of FLAG-HDA6 or 5 μM of His-HDA6) were incubated with 100 μM (for FLAG-HDA6) or 200 μM (for His-HDA6) of acetylated peptides in a reaction buffer containing 10 mM Tris–HCl buffer (pH 8), 150 mM NaCl and 10% glycerol at 30°C for 3 h. The reactions were stopped by adding a one-third reaction volume of 20% TFA and immediately frozen in liquid N2. Samples were then analyzed by LC–MS with a Vydac 218TP C18 column (4.6 mm × 250 mm, 5 μm; Grace Davison, Columbia, MD, Cat# 75799-130).

### Histone protein purification and western blot

Total histone proteins were extracted with a Histone Extraction kit (Abcam, Cat# AB113476). Briefly, 100 mg of 12-day-old Arabidopsis seedlings were quickly frozen in liquid nitrogen and ground to a powder with steel beads in 1.5 ml Eppendorf safe-locked tubes by shaking. Then 500 μl of 1× Pre-Lysis Buffer was added and thoroughly mixed with the ground sample. The mixture was centrifuged at 8000 × g for 1 min at 4°C. The pellet was then resuspended with 3 volumes (300 μl) of Lysis Buffer and incubated on ice for 30 min. After centrifugation at 11 000 × g for 5 min at 4°C, the supernatant was transferred into a new tube, and then 0.3 volume of the Balance-DTT Buffer was added quickly. The resulting protein solution was used for western blot assay with anti-H3 antibodies (Abcam, Cat# AB1791), anti-H3K9ac antibodies (Abcam, Cat# AB10812) and anti-H3K18ac antibodies (Millipore Sigma, Cat# 07-354).

### Total RNA isolation and Oxford Nanopore Technologies Direct RNA Sequencing (ONT DRS) library preparation, sequencing and data analysis

One hundred milligrams of 12-day-old Arabidopsis seedlings were ground into powder with two steel beads in 1.5 ml Eppendorf safe-locked tubes by shaking after fast-freezing with liquid nitrogen. Then 1 ml of Transzol (TransgenBiotech, Cat# ET101-01) was added to each sample and mixed well. After a 5-min incubation at room temperature, 200 μl of chloroform was added to the mixture. After shaking by hand for 30 s, the mixture was incubated at room temperature for another 3 min. Then the mixture was centrifuged at 10 000 × g for 15 min at 4°C. The colorless phase was transferred into a new tube, and 500 μl of isopropanol was added and mixed well. After incubation at room temperature for 10 min, the mixture was centrifuged at 4°C at 10 000 × g for 10 min. The acquired pellet was washed with 1mL of 75% ethanol. After removing as much of the ethanol as possible, the pellet was dissolved in 100 μl of RNase-free water. The extracted total RNA was then incubated with 5μl DNaseI (NEB, Cat# M0303S) and the DNase I buffer according to the manufacturer's instruction. The total RNA was then extracted with equal amount of acidic phenol (phenol:chloroform:isoamy 125:24:1, pH 4.5), rigorously mixed for 15 seconds and centrifuged at 4°C at 12 000 × g for 15 min. The acquired aqueous phases were then mixed with equal amount of chloroform, and centrifuged at 4°C at 12 000 × g for 15 min, repeat once, and the RNA in the aqueous phase were precipitation with 2.5 M LiCl. After precipitated for 1 h at –20°C, the total RNA was pelleted by centrifugation at 12 000 × g at 4°C for 30 min. The purified total RNA was then subjected to mRNA isolation with the Dynabeads™ mRNA Purification Kit (Thermo Fischer Scientific, Cat# 61006). Around 900 ng mRNA was used for the direct RNA-seq library preparation using the Direct RNA Sequencing Kit (Oxford Nanopore Technologies, Cat# SQK-RNA002). The prepared library was loaded onto R9.4.1 flowcells (Oxford Nanopore Technologies, Cat#: FLO-MIN106D) and sequenced with the MinION sequencer (Oxford Nanopore Technologies). The base-calling was performed with the Guppy 4.0.15 software (Oxford Nanopore Technologies). The obtained sequences were aligned to the C24 genome (23) with these parameters: ax splice; uf; k14 with minimap2 (36). The gene expression levels at the DMR loci were then extracted by deepTools (33) and BEDTools (34).

## RESULTS

### The *hda6* mutation reversed the *ros1-1* mutant phenotype, leading to DNA hypomethylation

**Figure 2.**
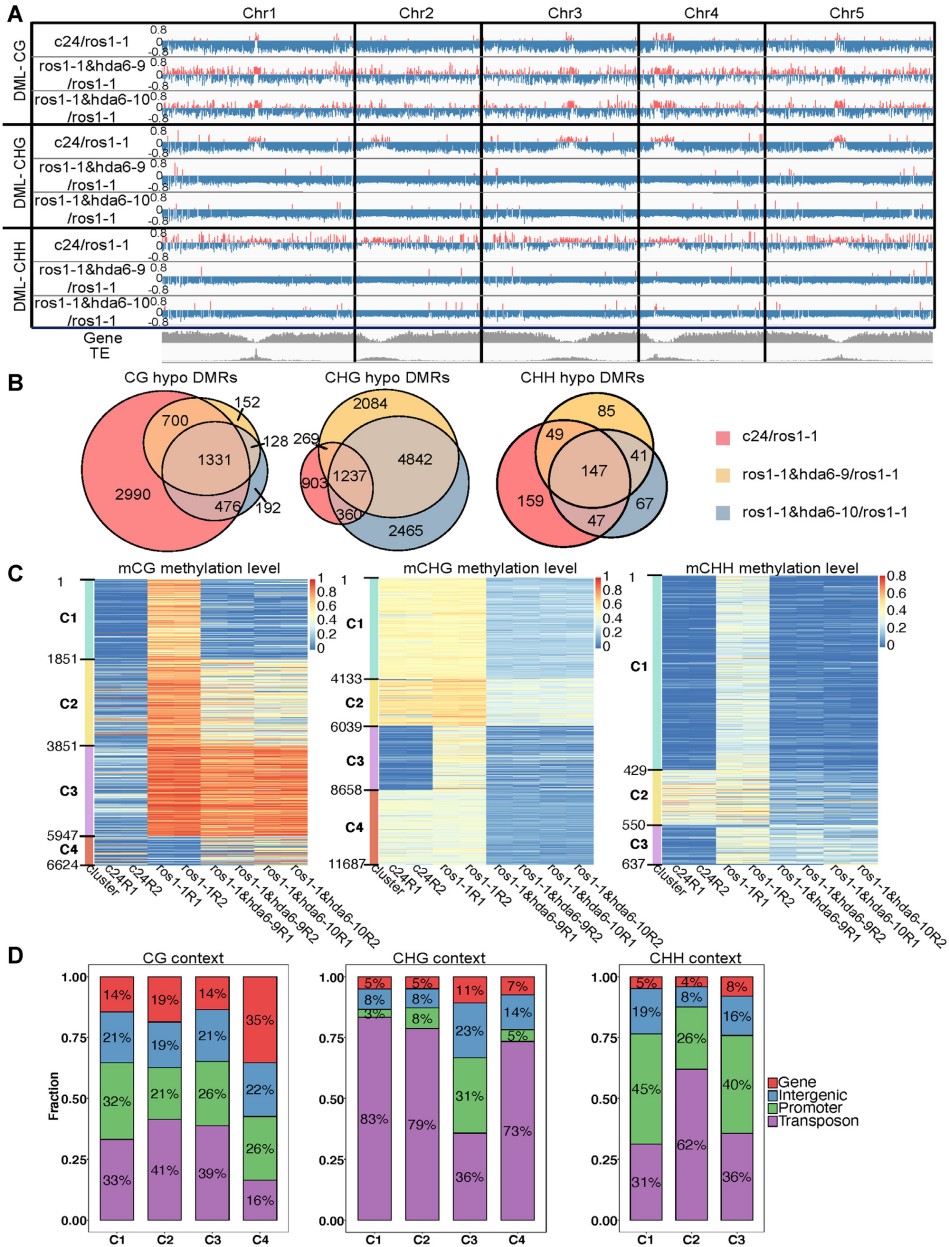
Genome-wide distributions of DNA methylation levels in Arabidopsis ecotype C24, *ros1-1*, *ros1-1&hda6-9*, and *ros1-1&hda6-10*. (**A**) An IGV plot showing the distributions of differentially methylated loci (DMLs) in C24/*ros1-1*, *ros1-1&hda6-9*/*ros1-1*, and *ros1-1&hda6-10*/*ros1-1* in the CG, CHG and CHH contexts along the five chromosomes, with the corresponding genic and transposable element (TE) regions at the bottom in grey. Red: hypermethylated DMLs; blue: hypomethylated DMLs. (**B**) Venn diagrams showing the overlapping hypo-DMRs (differentially methylated regions) among the C24/*ros1-1*, *ros1-1&hda6-9*/*ros1-1* and *ros1-1&hda6-10*/*ros1-1* comparisons in the CG, CHG and CHH contexts. (**C**) Heat maps of DMR clusters sorted according to their relative DNA methylation levels across the four genotypes. Each genotype is presented with two replicates (R1 and R2). (**D**) Proportions of the DMRs being located in each of the four genomic regions (gene, intergenic, promoter and transposon) within each cluster from (C) in the CG, CHG and CHH contexts respectively.

Since ROS1 is a DNA demethylase, a mutation in the *ROS1* gene leads to genome-wide DNA hypermethylation. To confirm that this phenotype of the *ros1-1* mutant can be reversed by a mutation in *HDA6* as previously reported (13,14), we performed whole-genome bisulfite sequencing in Arabidopsis wild-type C24, the *ros1-1* mutant and two *hda6* mutants constructed in the *ros1-1* background (*ros1-1&hda6-9* and *ros1-1&hda6-10*). Both the *ros1-1&hda6-9* and *ros1-1&hda6-10* mutants significantly reversed the hypermethylation caused by *ROS1* mutation in CG, CHG and CHH contexts (Figure 2A).

However, when comparing *ros1-1* to C24, we found the pericentromeric regions were hypomethylated, especially in the CHG context (Figure 2A). Similarly, when comparing *ros1-4* to Col-0, we also found hypomethylation in the pericentromeric region, and more pronounced in the CHG context as well (Supplementary Figure S1A). While a previous study (21) also showed that mutations in *ROS1* caused hypomethylated DMR, our work provides the first evidence to show the location of these regions. Our results suggested that other DNA demethylases, such as DME, may have been activated to compensate for the lack of ROS1 function in a site-specific context. The mutation of DME, which has also been reported to function at pericentromeric regions (8,9), in the *ros1-4* mutant background reverted the hypomethylated pericentromeric DNA back to wild-type levels (Supplementary Figure S1A) suggested that DNA hypomethylation at the pericentromeric regions in the *ros1* mutant was a consequence of the compensation by DME. Our results therefore demonstrated the compensation effects of DME to *ros1* mutant at the pericentromeric regions.

In both *ros1-1&hda6* double mutants (*ros1-1&hda6-9* and *ros1-1&hda6-10*), the genome-wide hypermethylation shown in the *ros1-1* mutant was reversed, resulting in even stronger hypomethylation at the pericentromeric regions, especially in the CHG context (Figure 2A). As discussed above, the loss of ROS1 activity was partially compensated by DME, most notably at the pericentromeric regions (8), and centered around long transposable elements (TEs) enriched with the H3K9me2 mark (9). The *hda6* mutation may have provided a more relaxed chromatin environment for DME, leading to more DNA demethylation at the pericentromeric regions.

We set the cut-offs for the differential methylation levels at ≥40%, ≥25% and ≥15% for the CG, CHG and CHH contexts, respectively, when mapping the differentially methylated regions (DMRs) between the *ros1-1* single mutant and the wild type (C24) or the *ros1-1&hda6* double mutants. Overall, the proportion of common CG-hypomethylated DMRs among those found in either *ros1-1&hda6-9/ros1-1* or *ros1-1&hda6-10/ros1-1* was much higher than that found in C24/*ros1-1* (Figure 2B, left panel). On the other hand, CHG-hypomethylated DMRs showed the opposite pattern (Figure 2B, middle panel). The two *ros1-1&hda6/ros1-1* comparisons showed around 10 times more unique CHG-hypomethylated DMRs than the number found in C24/*ros1-1*, suggesting that HDA6 activities may have prevented DNA demethylases to act on the DNA in the CHG context. Like in the CG context, the CHH-hypermethylated DMRs caused by the *ros1* mutation could also be reverted to normal methylation levels in the *ros1-1&hda6* double mutants (Figure 2B, right panel). It is worthwhile noting that the *hda6* mutation-specific CHH-hypomethylated DMRs in Cluster 2 mostly occurred in the TE regions (Figure 2C and D, right panel). These results indicate that other DNA demethylases may also be regulated through HDA6 activities since *ros1* mutation activated other DNA demethylases via increased H3K18ac in the *hda6* mutant. Moreover, the cluster analysis of CHG-hypomethylated DMRs revealed the common targets between ROS1 and HDA6, the majority of which was located in the promoter and intergenic regions (Cluster 3, Figure 2C and D, middle panels). This is consistent with previous reports on the role of ROS1 in preventing the heterochromatin spread. However, overall speaking, most of the *hda6* mutation-associated CHG-hypomethylated DMRs were found at the TE regions (Figure 2D, middle panel), supporting the observation that histone acetylation is crucial for DNA demethylase activities since DNA demethylases require acetylated H3K18ac (15,16). To confirm these findings, we compared the DNA methylation patterns between the *hda6-6* and *hda6-7* single mutants and their wild-type Col-0 parent, the WGBS dataset of which are publicly available (37) using our analytical pipeline. We found similar distribution and clustering patterns of DMRs in the comparisons between either of the two *hda6* single mutants and Col-0 as those found in the comparisons between *ros1-1* and C24 or the *ros1-1&hda6* double mutants (Supplementary Figure S2A–D). Together, these results demonstrated that HDA6 was crucial for maintaining CHG methylation in the TE regions, and its deacetylation activities then blocked DNA demethylases (e.g. DME) from accessing methylated CHG (Figure 2A and Supplementary Figure S1A).

**Figure 3.**
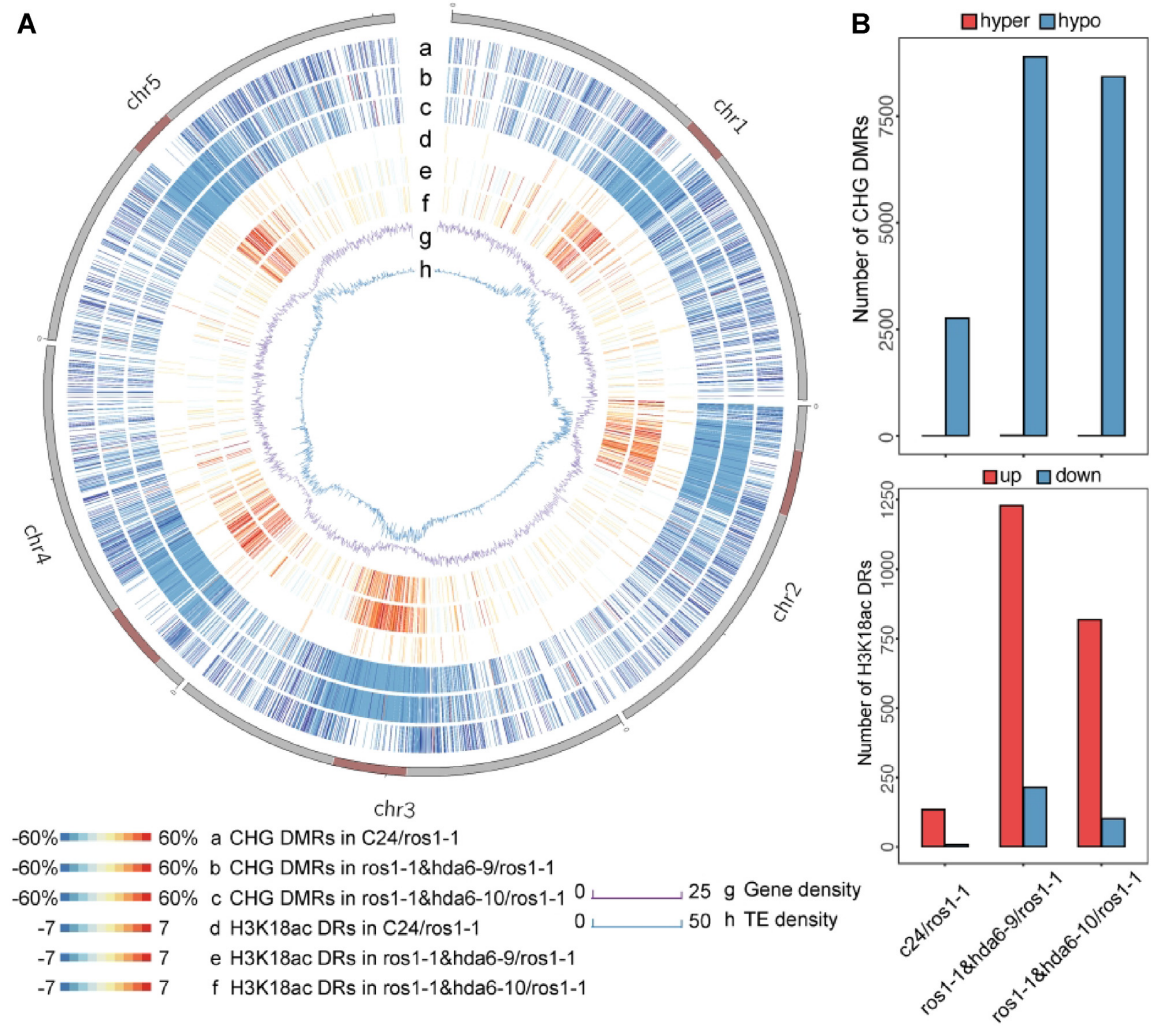
Genome-wide distributions of DMRs and differential H3K18ac accumulation regions at the CHG context. (**A**) A Circos plot showing the distributions of DMRs in the CHG context between C24/*ros1-1* (a), *ros1-1&hda6-9*/*ros1-1* (b), and *ros1-1&hda6-10*/*ros1-1* (c), and the differential H3K18ac accumulation between C24/*ros1-1* (d), *ros1-1&hda6-9*/*ros1-1* (e), and *ros1-1&hda6-10*/*ros1-1* (f), with the corresponding gene densities (g) and transposable element (TE) densities (h). The dark red bars on the chromosomes indicate centromeric regions. (**B**) Upper panel: numbers of DMRs in C24/*ros1-1*, *ros1-1&hda6-9*/*ros1-1* and *ros1-1&hda6-10*/*ros1-1*; lower panel: numbers of differential H3K18ac peaks in C24/*ros1-1*, *ros1-1&hda6-9*/*ros1-1* and *ros1-1&hda6-10*/*ros1-1*.

Interestingly, CHG-hypomethylated DMRs, but not CG- or CHH-hypomethylated DMRs, were clustered at the pericentromeric regions when comparing *ros1-1&hda6-9* or *ros1-1&hda6-10* to *ros1-1*. This pattern was not observed when comparing wild-type C24 to *ros1-1* (Figure 3A, Supplementary Figure S3B and S4B), implying that HDA6 mainly targets TEs and repeat sequences concentrated in the pericentromeric regions (38). To further confirm that the DMRs in pericentromeric regions were caused by the loss of HDA6 function, we performed the same analysis with the two *hda6* single mutants, *hda6-6* and *hda6-7* (37), as we did for the *ros1-1&hda6* double mutants. Similar patterns of CHG hypomethylation in the pericentromeric regions were observed in the *hda6-6* and *hda6-7* single mutants compared to the wild-type (Supplementary Figure S1B). Therefore, we can conclude that the decrease in CHG methylation in the pericentromeric regions in the *ros1-1&hda6* double mutants was caused by DME activities and the loss of HDA6 function.

### HDA6 is an eraser of the histone mark H3K18ac

Both *ros1-1&had6-9* and *ros1-1&hda6-10* had lower methylation levels compared to *ros1-1*, and the *hda6-6* and *hda6-7* single mutants also exhibited a similar hypomethylation pattern when compared to the wild-type Col-0, particularly in the CHH and CHG contexts (Figure 2A and Supplementary Figure S2A). Although DNA demethylases were first identified decades ago (39,40), the mechanism(s) of DNA demethylation have only just been recently unveiled (15,18,19). The H3K18ac mark is a prerequisite for DNA demethylation (15,16). Hence, it is possible that HDA6 may be instrumental in maintaining CHG methylation via its deacetylation activity on histone H3K18ac. To test this hypothesis, we performed ChIP-Seq experiments on *ros1-1& hda6-9* and *ros1-1& hda6-10*, *ros1-1* and the wild-type C24 using H3K18ac-specific antibodies. In comparing between the double mutants and *ros1-1*, the differential peaks of H3K18ac accumulation were mainly concentrated in the pericentromeric regions of all five chromosomes (Figure 3A, rings e and f), and these locations corresponded to the CHG-hypomethylated regions identified in the two *ros1-1&hda6* mutants (Figure 3A, rings b and c). In line with the ChIP-seq results, the H3K9ac and H3K18ac levels were found to be slightly increased in the *ros1-1&hda6-9* and *ros1-1&hda6-10* double mutants compared to those in *ros1-1* (Supplementary Figure S5), supporting the hypothesis that HDA6 serves as an H3K18ac eraser at the pericentromeric heterochromatin regions.

**Figure 4.**
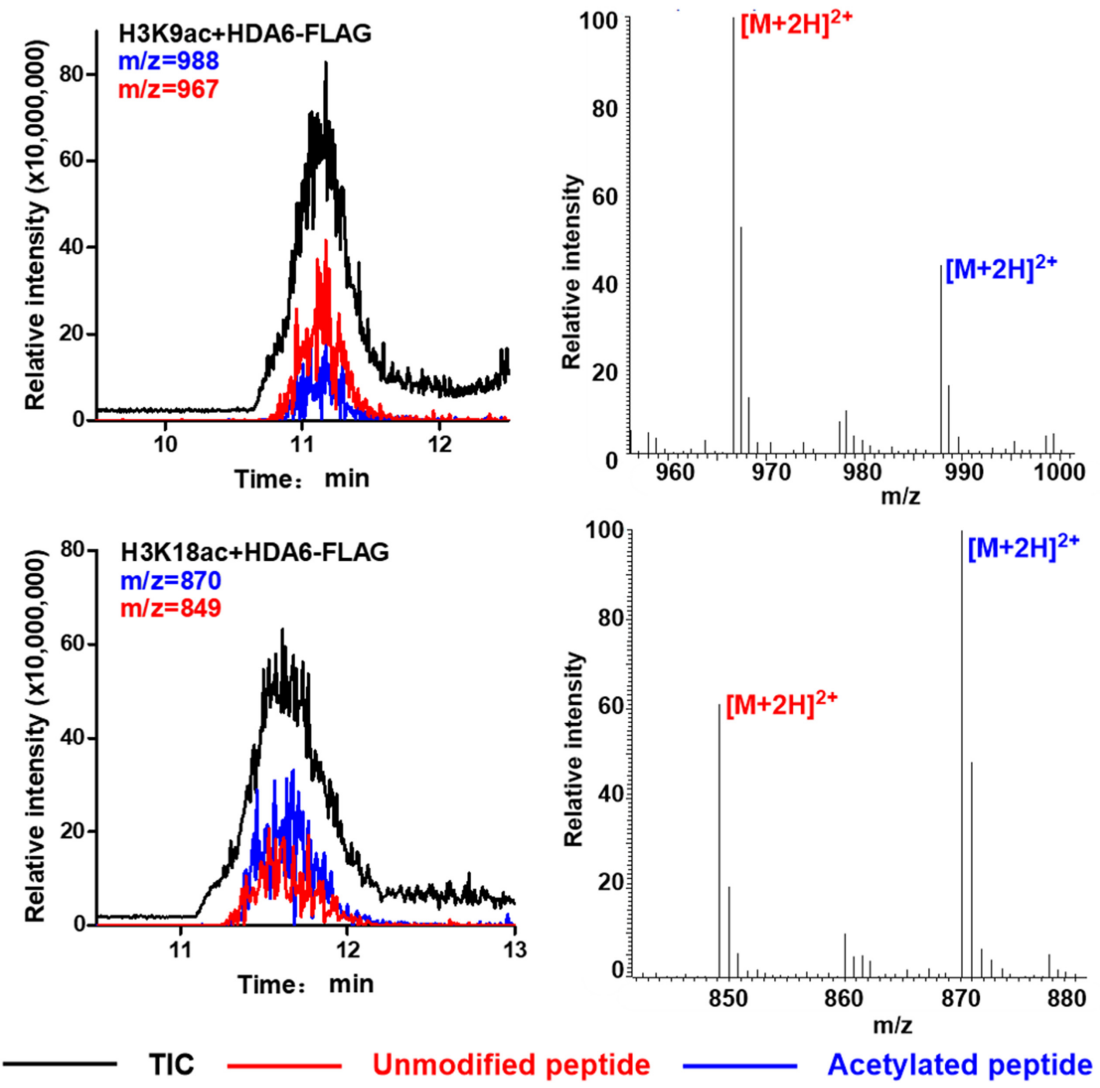
HDA6 can effectively deacetylate H3K9ac and H3K18ac *in vitro*. The hydrolysis of acetylated peptides by FLAG-tagged HDA6 was analyzed by LC–MS. Black traces show the total ion intensity for all ion species with *m/z* ranging from 300 to 2000; red traces show the ion intensity for the mass of unmodified (deacetylated) peptides; and blue traces show the ion intensity for the mass of acetylated peptides. TIC: total ion count.

The eraser activity of HDA6 was confirmed with the purified recombinant full-length HDA6 protein and its mutated version (constructed from the cDNAs of *hda6-9* mutant allele). The wild-type HDA6 protein purified from *E. coli* exhibited deacetylation activities towards H3K9ac and H3K18ac, whereas the mutant HDA6 proteins showed no deacetylase activity (Supplementary Figure S6). On the other hand, 3xFLAG-tagged full-length HDA6 purified from stable transgenic *A. thaliana* showed high deacetylase activities on H3K18ac and H3K9ac (Figure 4). This observation is similar to that reported for the mammalian homolog of HDA6, in which only the recombinant proteins expressed in eukaryotic cells showed deacetylase activities (41). These results indicated that HDA6 proteins expressed in eukaryotic systems may have additional posttranslational modifications that enhance the deacetylation activity on H3K18ac. A recent report showed that phosphorylation of the serine residue S427 of HDA6 was crucial for its enzymatic function (42).

### HDA6 is crucial for chromatin transition and silencing

**Figure 5.**
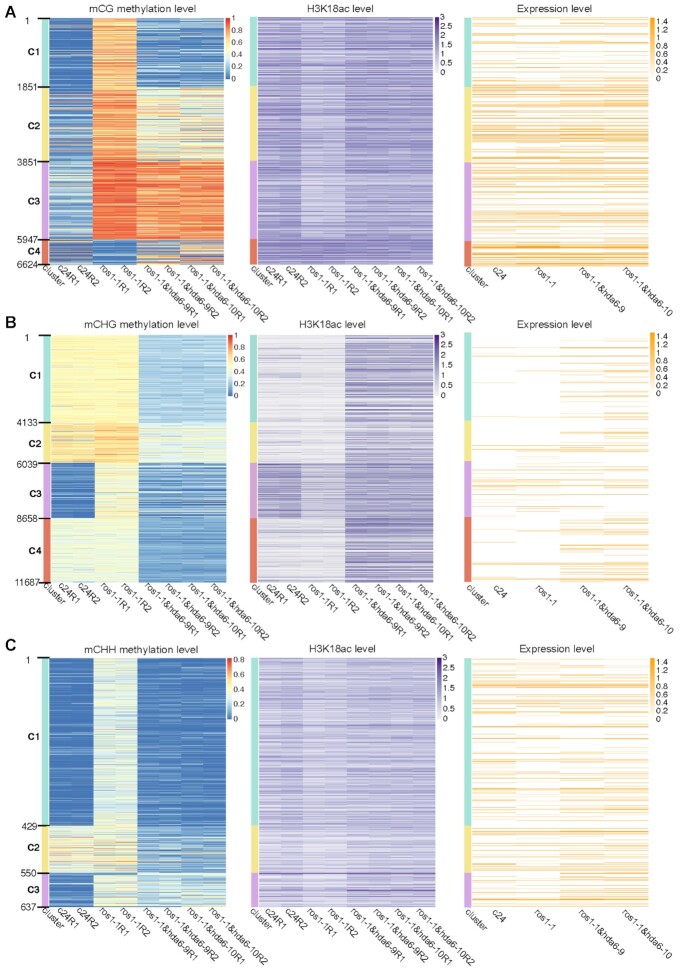
DNA methylation, histone H3K18ac accumulation and gene expression patterns of clustered DMRs in the CG (**A**), CHG (**B**) and CHH contexts (**C**). DMRs were clustered according to their relative DNA methylation levels across the four genotypes (C24, *ros1-1*, *ros1-1&hda6-9* and *ros1-1&hda6-10*). Each genotype is represented by two biological replicates (R1 and R2).

Previous studies on *hda6-6* and *hda6-7* showed that the loss-of-function of HDA6 not only affected nucleolar dominance (43) and activated the nucleolus organizer region (NOR) (44), but it also activated the constitutive pericentromeric heterochromatin regions (13,45). These previous reports identified histone deacetylation by HDA6 as the main mode of preventing the activation of heterochromatin regions, without clearly pinpointing the target for this enzyme. In this study, we identified H3K18ac to be a crucial target of HDA6 in maintaining CHG methylation at pericentromeric regions. To further explore whether H3K18ac accumulation and CHG methylation could prevent the activation of heterochromatin, we used Oxford Nanopore Technologies Direct RNA Sequencing (ONT DRS) (46) to profile the activated TEs and repeat sequences in the constitutive heterochromatin regions in both *ros1-1&hda6* double mutants. Unlike next-generation sequencing (NGS), ONT DRS can directly sequence native, full-length RNA molecules without requiring them to be first converted into cDNAs, and it is therefore not constrained by the size capabilities of commercially available reverse transcriptase (47). Thus this sequencing technology can detect the highly repetitive sequences in the heterochromatin regions (48). Our results showed the distribution patterns of DMRs were correlated with the presence of histone H3K18ac and gene expression levels in the CHG (Figure 5B) and CHH contexts (Figure 5C), but not in the CG context (Figure 5A). We have already shown that H3K18ac accumulation was highly correlated with CHG methylation (Figure 3A) and partially correlated with CHH methylation (Supplementary Figure S4), but had no obvious correlation with CG methylation (Supplementary Figure S3). These results indicated that CHG methylation and H3K18ac deacetylation are highly correlated and could affect expressions of the corresponding genomic regions.

**Figure 6.**
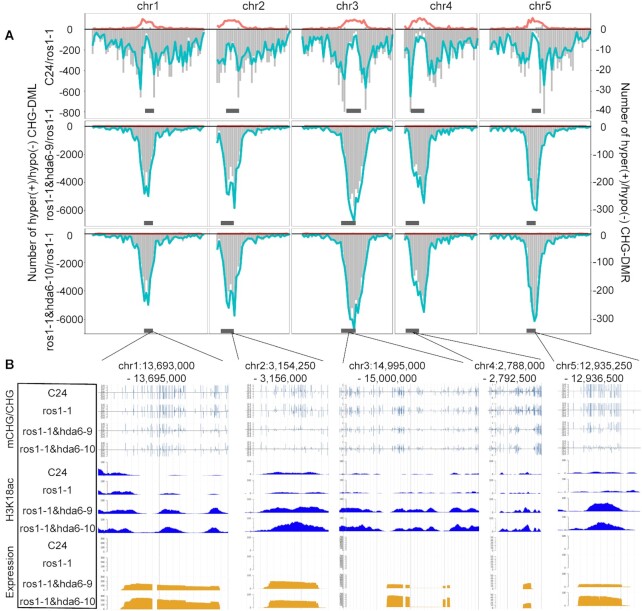
HDA6 increased DNA methylation, suppressed histone H3K18ac accumulation and reduced transcript abundance in the centromeric regions in the CHG context. (**A**) Numbers of DMLs or DMRs in the CHG context along all five chromosomes. Horizontal grey bars at the bottom indicate centromeric regions. Turquoise lines represent the distribution of hypomethylated loci; red lines represent the hypermethylated loci; vertical grey bars represent the hyper-/hypo- DMRs. (**B**) Representative sections from each chromosomal centromeric region showing the DNA methylation levels, H3K18ac accumulation and transcript abundance in the CHG context in each genotype.

To further investigate the effects of CHG methylation and H3K18ac accumulation on expressions in the pericentromeric region, we listed all activated transcripts in the pericentromeric regions *in ros1-1&hda6* double mutants (Supplementary Table S1). To illustrate the relationship between gene expression in activated pericentromeric regions and H3K18ac accumulation and CHG hypomethylation, we used J-browse to snapshot some examples from each pericentromeric region of all five chromosomes (Figure 6B). All the examples support the notion that HDA6 malfunction promoted H3K18ac accumulation, and hypomethylation at CHG context in pericentromeric regions. These data further supported the notion that HDA6 plays important roles in maintaining the heterochromatin status.

## DISCUSSION

### HDA6 maintains DNA methylation via its deacetylation activity on H3K18ac

HDA6 has been confirmed to mediate DNA methylation at the CG and CHH contexts via its direct interaction with DNA methyltransferase I (MET1) (49) and its involvement in the RNA direct DNA methylation (RdDM) pathway (14,50). Although HDA6 has been frequently identified in different forward genetic screening systems (13,14,50–53), it is mainly regarded as an epigenetic regulator to suppress reporter gene expression (51–53). Our whole-genome bisulfite sequencing data showed that HDA6 not only partially antagonized the ROS1-mediated DNA demethylation at the CHH and CG contexts, but it also played a more central role in CHG methylation at the pericentromeric regions. Our results also suggested that other DNA demethylases, e.g. DEMETER, DML2 and DML3, might share some common targets with ROS1, and that these enzymes require H3K18ac for their demethylase activities, particularly in the CHG context in the pericentromeric regions, since the hda6 mutation resulted in CHG hypomethylation in these regions. Meanwhile DME has been reported to demethylate DNA at the pericentromeric regions (8,9), and the *dme* mutation could reverse the CHG hypomethylation caused by the *ros1* mutation (Supplementary Figure S1A). These results clearly showed that CHG demethylation at the pericentromeric regions was mediated by DME, and that HDA6 maintained CHG methylation by preventing DNA from being demethylated by DME.

Since HDA6 has been reported to directly interact with MET1 (49) and SUVH4/5/6 (42). The SUVH4/5/6 catalyzed H3K9me2 can then recruit CMT3 (11). It was thought that HDA6 can directly recruit MET1 to promote DNA methylation (49,54) or indirectly recruit CMT3 to maintain CHG methylation (11,42,55). However, our results suggested a different mechanism for maintaining DNA methylation at the CHG context, that MET1 recruits HDA6 to the methylated regions, where HDA6 can deacetylate H3K18ac and H3K9ac, and further recruit SUVH4/5/6 to form repressive epigenetic mark H3K9me2 to prevent DNA demethylases from accessing their targets.

### HDA6 provides the missing link between DNA methylation and histone modifications

Our data confirmed that HDA6 is an eraser of histone acetylation, H3K18ac and H3K9ac, which are crucial markers for DNA demethylation (Figure 1). The deacetylation of H3K9 is necessary for SUVH4/5/6 to add dimethyl groups to form the repressive H3K9me2 mark (56). Once the H3K9me2 marks are deposited, the facultative heterochromatin is transformed into constitutive heterochromatin (Figure 1). However, in the *hda6* mutants, the IDM1-acetylated H3K18ac and HAG1-acetylated H3K9ac cannot be deacetylated. As a consequence, the dimethylation of H3K9 by SUVH4/5/6 was limited, resulting in fewer repressive H3K9me2 marks being deposited in the pericentromeric regions. The direct interaction between HDA6 and SUVH4/5/6 (42) can block the IDM1 access to H3K18, and consequently prevent DNA demethylation in heterochromatin regions. While, the H3K18ac accumulation in these regions (especially in the CHG context) can facilitate DNA demethylation by DNA demethylases. CMT3 interacts with H3K9me2-containing nucleosomes (11), which is also a target of DME (9). Hence it is possible that the binding of CMT3 to H3K9me2-containing nucleosomes quickly re-methylates DNA in the CHG context which has been demethylated by DME during chromatin remodeling mediated by H2A.W, linker histone H1 (55), or other chromatin remodellers to maintain the heterochromatin status (Figure 1).

## DATA AVAILABILITY

The WGBS data for Col-0 genetic background of the mutants used were downloaded from the GEO database (accession no. GSE39901 for the BS-seq of *hda6-6*, *hda6-7*, Col-0 for *hda6*, *SUVH4/5/6* and Col-0 (20); accession no. GSE83802 for WGBS data of *ros1-4* and Col-0 (21); accession no. GSE164916 for the WGBS data of *drdd^DD7 pro^* and Col-0 (10)).

Sequencing data generated in this study have been deposited with NCBI (https://www.ncbi.nlm.nih.gov/sra/PRJNA694533) under the accession number PRJNA694533. Gene accession numbers: *DME*, At5g04560; *ROS1*, At2g36490; *DML2*, At3g10010; *DML3*, At4g34060; *HDA6*, AT5G63110; *IDM1*, AT3G14980; *MET1*, AT5G49160; *DRM1*, AT5G15380; *DRM2*, AT5G14620; *CMT2*, AT4G19020; *CMT3*, AT1G69770; *SUVH4*, AT5G13960; *SUVH5*, AT2G35160; *SUVH6*, AT2G22740; *IBM1*, AT3G07610; *HAG1*, AT3G54610. Seed stock numbers: C24, CS24939; *ros1-1*, Germplasm:4010764159; *ros1-1&hda6-9*, CS72565; *ros1-1&hda6-10*, CS72566; HDA6-FL-YFP-3FLAG in Col-0, CS72567.

## Supplementary Material

gkab706_Supplemental_Files
