# Supplementary material for: AtHDA6 functions as an H3K18ac eraser to maintain pericentromeric CHG methylation in Arabidopsis thaliana
Source: Nucleic Acids Res. 2021 Aug 17;49(17):9755–67. doi: 10.1093/nar/gkab706 (PMC8464031; doi:10.1093/nar/gkab706)
Supplement: gkab706_Supplemental_Files [file gkab706_supplemental_files.zip › AtHDA6 Supplementary Figures-.pdf]

Supplementary materials : AtHDA6 functions as an H3K18ac eraser to maintain pericentromeric CHG methylation in *Arabidopsis thaliana*

Qianwen Wang[1,2,8], Xiucong Bao[3,8], Shengjie Chen[1,2,8], Huan Zhong[4,8], Yaqin Liu[1,2], Li Zhang[1,2], Yiji Xia[2,4,5], Friedrich Kragler[6], Ming Luo[7*], Xiang David Li[3*], Hon-Ming Lam[1,2*], Shoudong Zhang[1,2, 9*]

1. School of Life Sciences, The Chinese University of Hong Kong, Shatin, Hong Kong Special Administrative Region.
2. Center for Soybean Research of the State Key Laboratory of Agrobiotechnology, The Chinese University of Hong Kong, Shatin, Hong Kong Special Administrative Region.
3. Department of Chemistry, The University of Hong Kong, Pokfulam Road, Hong Kong Special Administrative Region.
4. Department of Biology, Hong Kong Baptist University, Kowloon, Hong Kong Special Administrative Region.
5. State Key Laboratory of Environmental and Biological Analysis, Hong Kong Baptist University, Kowloon, Hong Kong Special Administrative Region.
6. Max-Planck-Institute of Molecular Plant Physiology, Wissenschaftspark Golm, Am Mühlenberg 1, 14476 Golm, Germany.
7. Agriculture and Biotechnology Research Center, Guangdong Provincial Key Laboratory of Applied Botany, Center of Economic Botany, Core Botanical Gardens, South China Botanical Garden, Chinese Academy of Sciences, Guangzhou 510650, China
8. These authors contributed equally
9. Lead contact

* To whom correspondence should be addressed. Tel:00852-39431270; Fax: 00852-39436336; Email: shoudongzhang@cuhk.edu.hk ; Correspondence may also be addressed to hon-ming@cuhk.edu.hk (H.-M. L.); xiangli@hku.edu.hk (X. D. L.) ; luoming@scbg.ac.cn (M. L.) .

The authors wish it to be known that, in their opinion, the first 4 authors should be regarded as joint First Authors.

Supplementary Table S1-S3

Table S1. List of transcripts that have higher expressions in the centromeric and pericentromeric regions of the *ros1-1&hda6* double mutants than in the wild type (C24) or *ros1-1* single mutant.

Table S2. Primers used in the study.

Table S3. Peptides used in the study.

Supplementary Figures S1-S6

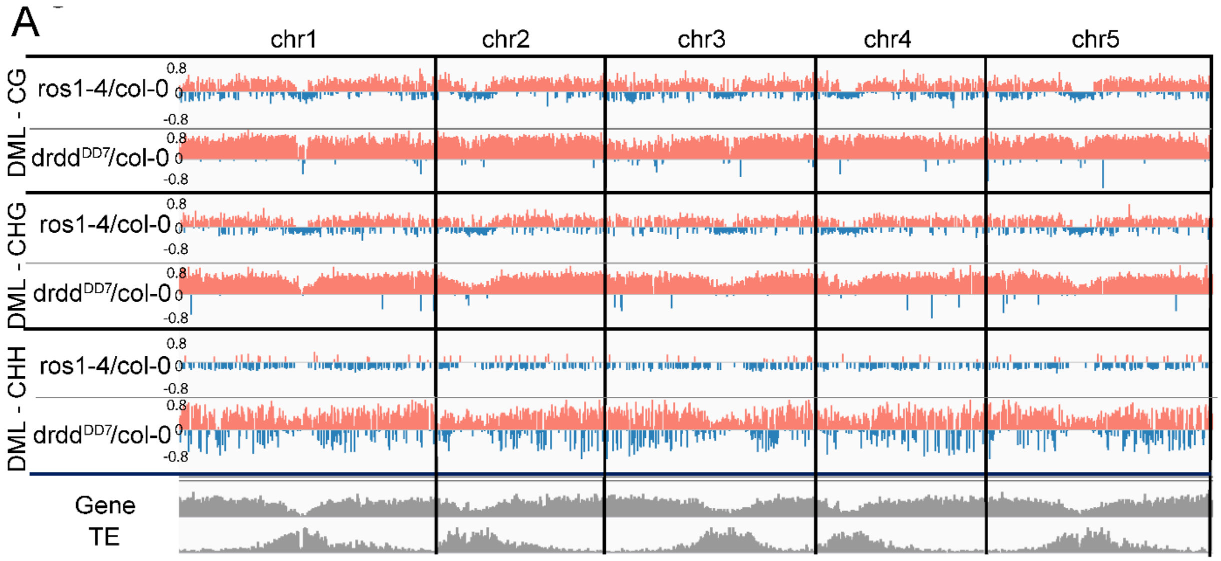

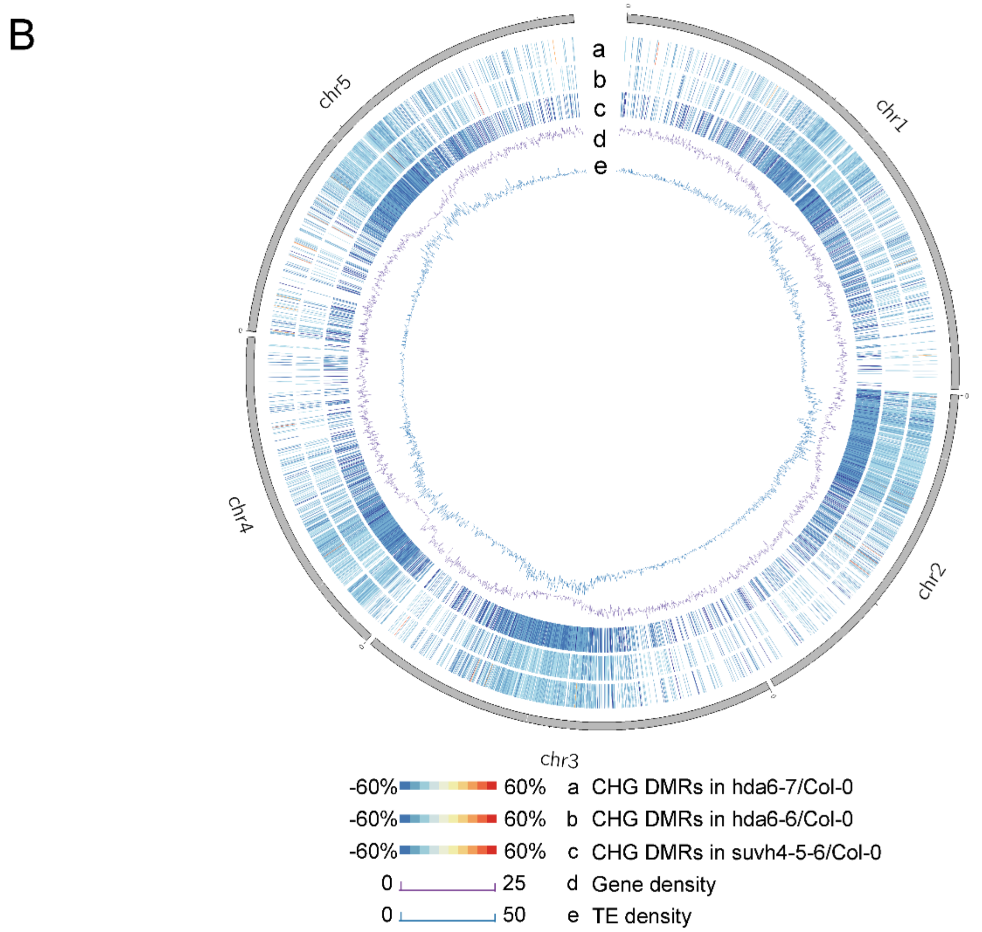

Figure S1. Distributions of differentially methylated loci (DMLs), differentially methylated regions (DMRs) in the Arabidopsis genome, between DNA demethylation-deficient mutants (*ros1-4* or *drdd*$^{DD7\ pro}$) and wild type (Col-0) and between DNA methylation-deficient mutants (*hda6-6*, *hda6-7* or *suvh4-5-6*) and wild type (Col-0). (A) An IGV plot of the distributions of DMLs of *ros1-4*/Col-0 and *drdd*$^{DD7\ pro}$/Col-0 in the CG, CHG and CHH contexts along the five chromosomes. Red: hypermethylation; blue: hypomethylation, compared to wild type. TE: transposable element. (B) A circos plot showing the distributions of DMRs of *hda6-7*/Col-0 (a), *hda6-6*/Col-0 (b), and *suvh4-5-6*/Col-0 (c) in the CHG context along the five chromosomes.

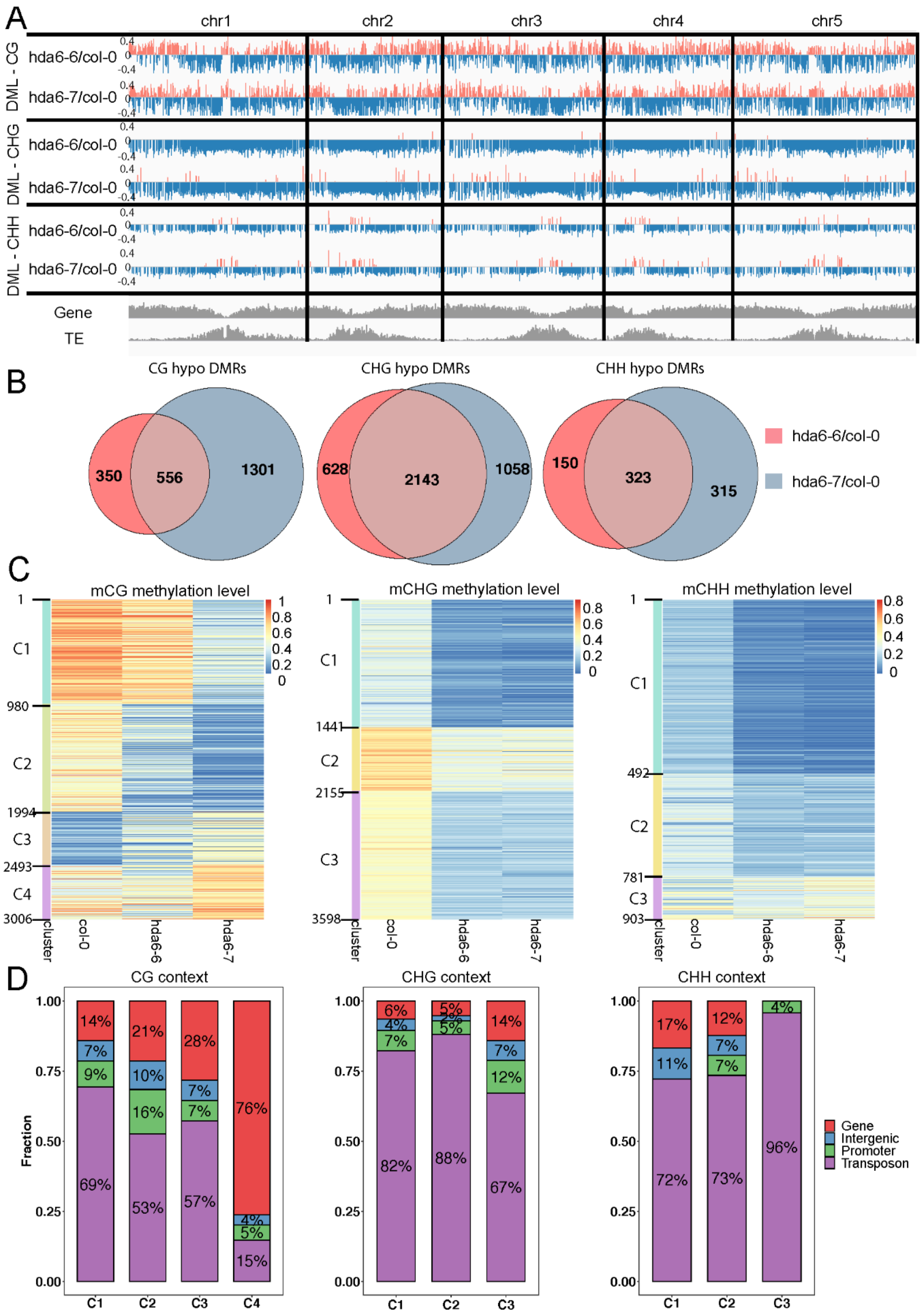
A
chr1
chr2
chr3
chr4
chr5
DML - CG
DML - CHG
DML - CHH
hda6-6/col-0
hda6-7/col-0
Gene
TE
B
CG hypo DMRs
CHG hypo DMRs
CHH hypo DMRs
350
556
1301
628
2143
1058
150
323
315
hda6-6/col-0
hda6-7/col-0
C
mCG methylation level
mCHG methylation level
mCHH methylation level
C1
C2
C3
C4
cluster
col-0
hda6-6
hda6-7
D
CG context
CHG context
CHH context
Fraction
Gene
Intergenic
Promoter
Transposon

Figure S2. Features of DNA methylation patterns in the Arabidopsis wild type (Col-0) compared to the DNA methylation-deficient mutants, *hda6-6*, and *hda6-7*. (A) An IGV plot of the distributions of DMLs of *hda6-6*/Col-0 and *hda6-7*/Col-0 in the CG, CHG, and CHH contexts along the five chromosomes. (B) Venn diagram**s** showing the numbers of hypo-DMRs of *hda6-6*/Col-0 and *hda6-7*/Col-0 and hypo-DMRs common to both sets of comparisons, in the CG, CHG and CHH contexts. (C) Heat maps of DMR clusters sorted according to their relative methylation levels in each genotype in the CG, CHG, and CHH contexts. (D) Proportions of the DMRs being located in each of the four genomic regions (gene, intergenic, promoter and transposon) within each cluster from (C) in the CG, CHG, and CHH contexts respectively.

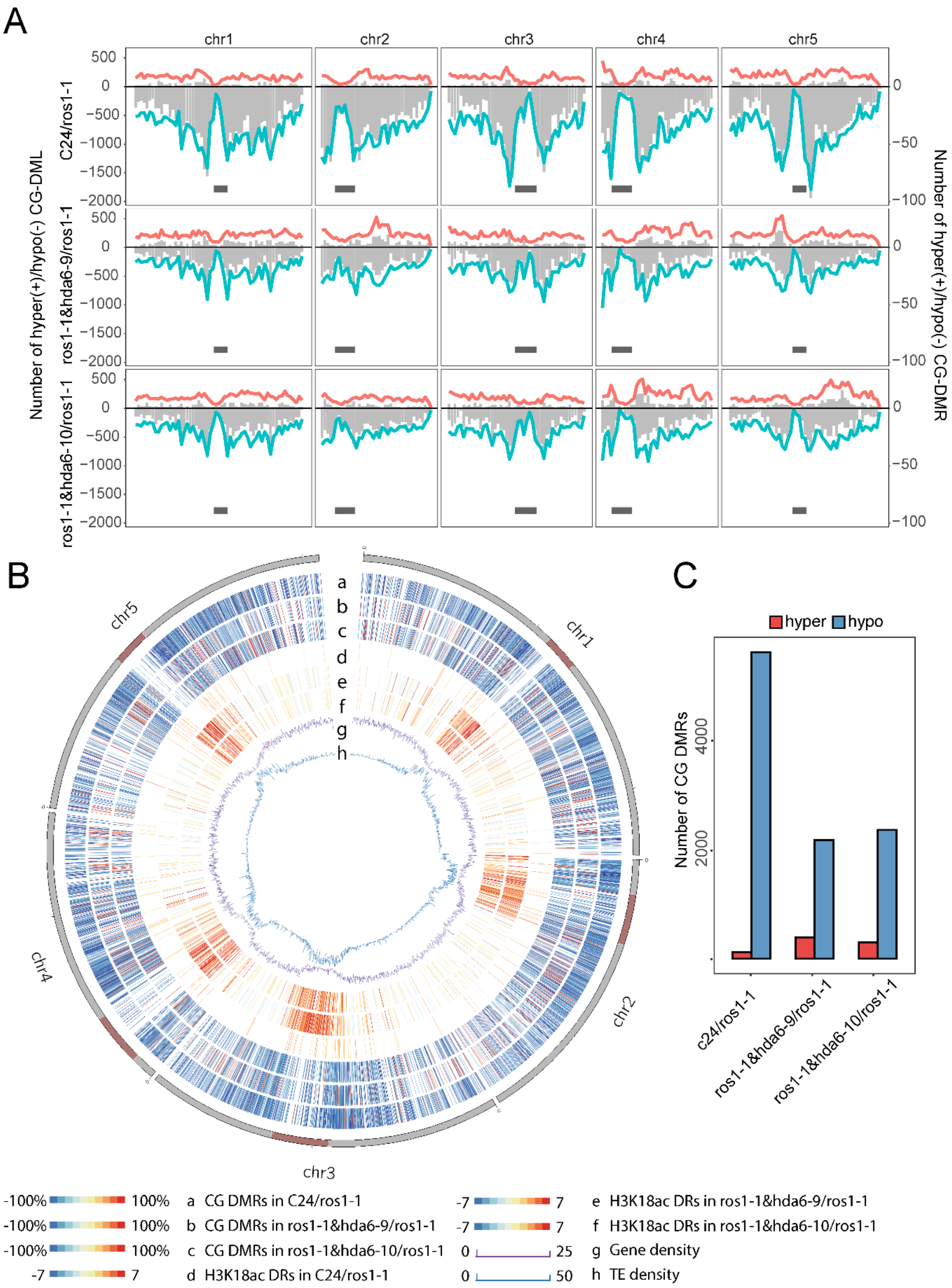
A
chr1
chr2
chr3
chr4
chr5
C24/ros1-1
ros1-1&hda6-9/ros1-1
ros1-1&hda6-10/ros1-1
Number of hyper(+)/hypo(-) CG-DML
Number of hyper(+)/hypo(-) CG-DMR
500
0
−500
−1000
−1500
−2000
0
−50
−100
B
a
b
c
d
e
f
g
h
C
hyper
hypo
Number of CG DMRs
2000
4000
c24/ros1-1
ros1-1&hda6-9/ros1-1
ros1-1&hda6-10/ros1-1
-100% 100% a CG DMRs in C24/ros1-1
-100% 100% b CG DMRs in ros1-1&hda6-9/ros1-1
-100% 100% c CG DMRs in ros1-1&hda6-10/ros1-1
-7 7 d H3K18ac DRs in C24/ros1-1
-7 7 e H3K18ac DRs in ros1-1&hda6-9/ros1-1
-7 7 f H3K18ac DRs in ros1-1&hda6-10/ros1-1
0 25 g Gene density
0 50 h TE density

Figure S3. Distributions of DMLs and DMRs and differential H3K18ac accumulation along the Arabidopsis chromosomes in the CG context. (A) DMLs of C24/*ros1-1*, *ros1-1&hda6-9/ros1-1* and *ros1-1&hda6-10/ros1-1* along the chromosomes in the CG context. Horizontal grey bars at the bottom indicate centromeric regions. Turquoise lines represent the distribution of hypomethylated loci; red lines represent the hypermethylated loci; vertical grey bars represent the hyper-/hypo- DMRs. (B) A Circos plot showing the distributions of DMRs in the CG context in C24/*ros1-1* (a), *ros1-1&hda6-9/ros1-1* (b), and *ros1-1&hda6-10/ros1-1* (c), and the differential H3K18ac accumulation in C24/*ros1-1* (d), *ros1-1&hda6-9/ros1-1* (e), and *ros1-1&hda6-10/ros1-1* (f), with the corresponding gene densities (g) and transposable element (TE) densities (h). Red section in each chromosome represents the centromeric region. (C) Numbers of hypermethylated (red bars) and hypomethylated (blue bars) DMRs in the CG context in C24/*ros1-1*, *ros1-1&hda6-9/ros1-1* and *ros1-1&hda6-10/ros1-1*.

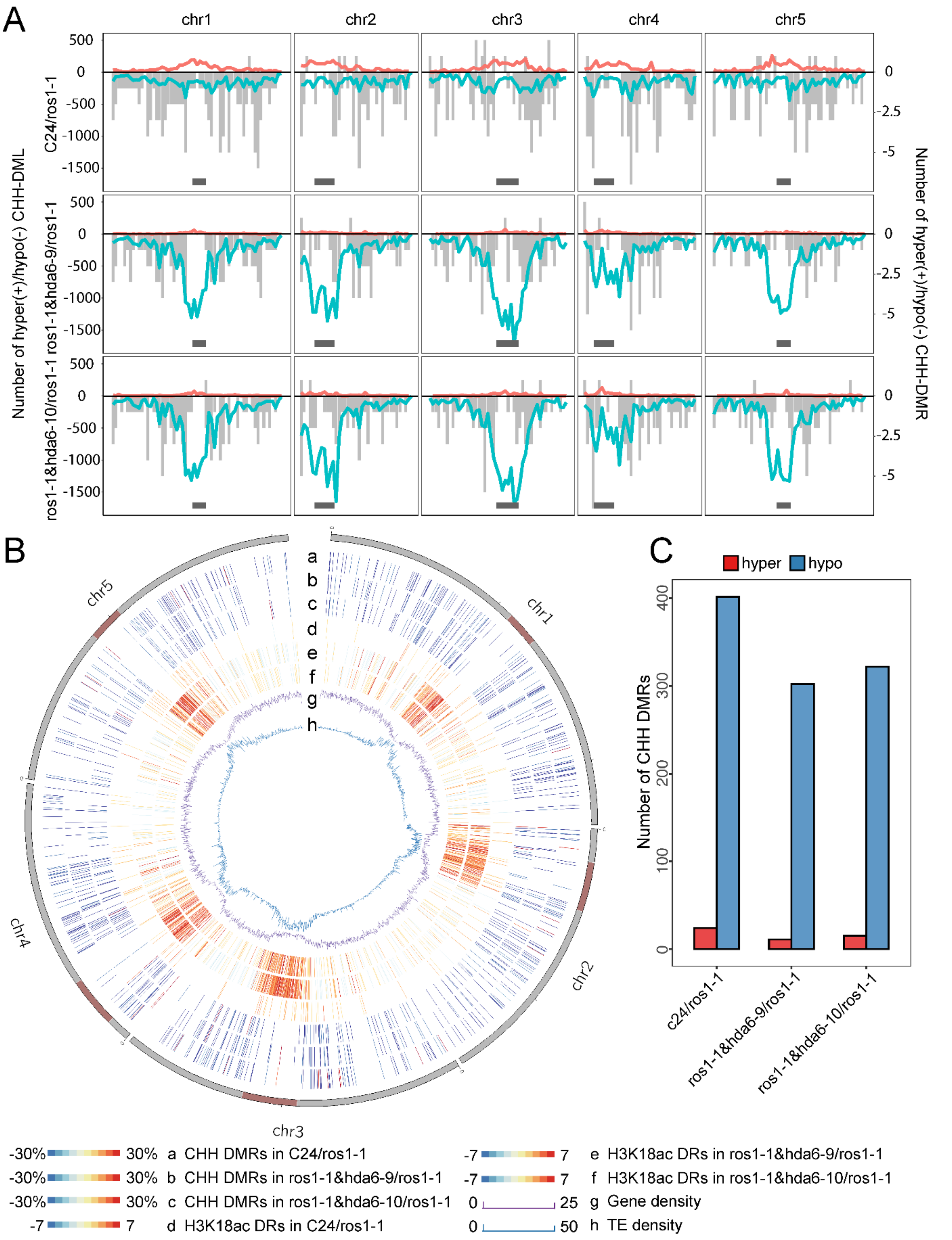

Figure S4. Distributions of DMLs and DMRs and differential H3K18ac accumulation along the Arabidopsis chromosomes in the CHH context. (A) DMLs of C24/*ros1-1*, *ros1-*

1&hda6-9/ros1-1 and *ros1-1&hda6-10/ros1-1* along the chromosomes in the CHH context. Horizontal grey bars at the bottom indicate centromeric regions. Turquoise lines represent the distribution of hypomethylated loci; red lines represent the hypermethylated loci; vertical grey bars represent the hyper-/hypo- DMRs. (B) A Circos plot showing the distributions of DMRs in the CHH context in C24/*ros1-1* (a), *ros1-1&hda6-9/ros1-1* (b), and *ros1-1&hda6-10/ros1-1* (c), and differential H3K18ac accumulation in C24/*ros1-1* (d), *ros1-1&hda6-9/ros1-1* (e), and *ros1-1&hda6-10/ros1-1* (f), with the corresponding gene densities (g) and transposable element (TE) densities (h). Red section in each chromosome represents the centromeric region. (C) Numbers of hypermethylated (red bars) and hypomethylated (blue bars) DMRs in the CHH context in C24/*ros1-1*, *ros1-1&hda6-9/ros1-1* and *ros1-1&hda6-10/ros1-1*.

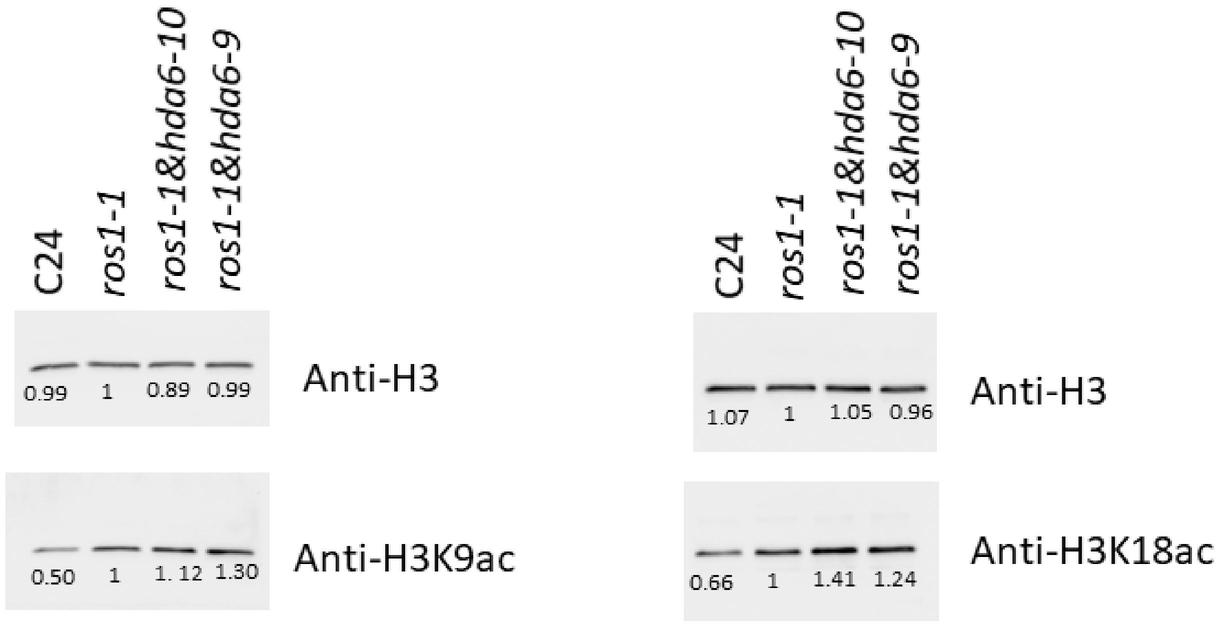

Figure S5. Western blot demonstrating HDA6 activities on H3K9ac and H3K18ac *in vivo*. H3 was used as a loading control. There was enhanced H3K9ac and H3K18ac accumulation in the double and single mutants over wild type (C24), and the acetylation levels are higher in both the double mutants than in *ros1-1*. Numbers below each band represent relative intensities, with that of *ros1-1* set to 1.

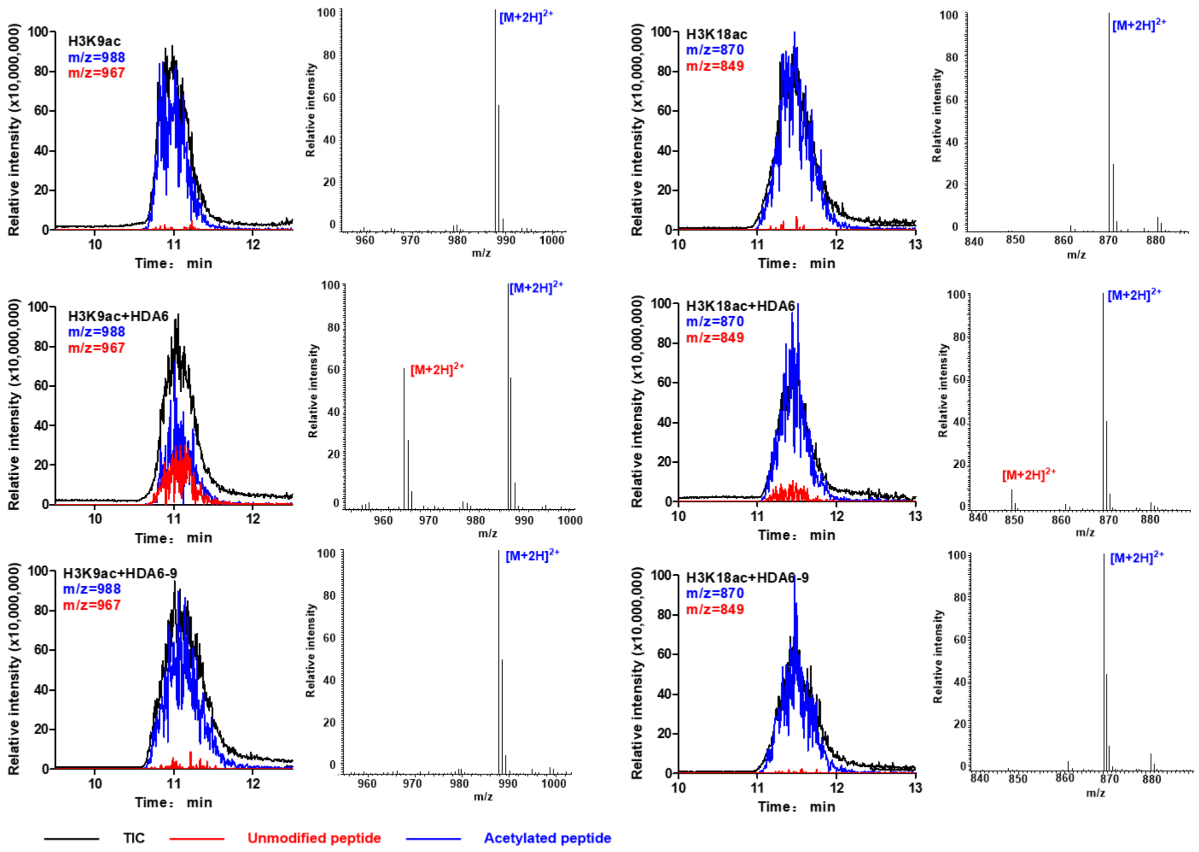

Figure S6. Results of in-vitro activity assay for recombinant HDA6 and its mutated versions purified from E. coli. The hydrolysis of the acetylated peptides by recombinant HDA6 and its mutated versions was analyzed by LC-MS. Black traces show total ion intensity (total ion counts, TIC) for all ion species with m/z from 300 to 2000; red traces show ion intensity for the masses of unmodified (deacetylated) peptides; and blue traces show ion intensity for the masses of acetylated peptides.
